# Does Antrum Size Matter in Sleeve Gastrectomy? Volume II—A Retrospective Multicentric Study with Long-Term Follow-Up

**DOI:** 10.3390/jcm13133912

**Published:** 2024-07-03

**Authors:** Claudio Gambardella, Simona Parisi, Salvatore Tolone, Francesco Saverio Lucido, Gianmattia del Genio, Luigi Brusciano, Rosetta Esposito, Domenico de Vito, Ludovico Docimo, Francesco Pizza

**Affiliations:** 1 Division of General, Oncological, Mini-Invasive and Obesity Surgery, University of Study of Campania “Luigi Vanvitelli”, 80131 Naples, Italy; simona.parisi@unicampania.it (S.P.); salvatore.tolone@unicampania.it (S.T.); francescosaverio.lucido@unicampania.it (F.S.L.); gdg@doctor.com (G.d.G.); luigi.brusciano@unicampania.it (L.B.); rosetta.esposito@unicampania.it (R.E.); domenico.devito@unicampania.it (D.d.V.); ludovico.docimo73@gmail.com (L.D.); 2 Department of Surgery, Aslnapoli2nord, Hospital “A. Rizzoli”, 80076 Naples, Italy; francesco_pizza@libero.it

**Keywords:** sleeve gastrectomy, antral preservation antral resection, GERD, weight loss

## Abstract

**Background:** Laparoscopic sleeve gastrectomy (LSG) is the most widespread bariatric procedure due to its safety and efficacy. Despite continuous refinement, achieving a globally standardized procedure remains challenging. Moreover, due to its wide adoption, numerous studies have focused on complications associated with the technique, such as gastroesophageal reflux disease (GERD). This study evaluates the impact of antrum size (wide antrectomy versus small antrectomy) in LSG on long-term anthropometric outcomes and complications in patients with morbid obesity. **Methods:** Body mass index (BMI), percentage of excess weight loss (%EWL) at a 5-year follow-up, GERD Health-Related Quality-of-Life (GERD-HRQL) scores, and obesity-related diseases of patients undergoing LSG with gastric resections starting 2 cm and 6 cm from the pylorus were retrospectively evaluated. **Results:** Between January 2015 and November 2019, 597 patients who met the criteria for LSG were included in the study. Group A (241 patients) underwent wide antrectomy, while Group B (356 patients) underwent small antrectomy. Weight, BMI, %EWL, and %TWL significantly improved at 6 and 12 months in the wide-antrectomy group. However, these differences diminished by 24 months, with no significant long-term differences in weight loss outcomes between the two groups at 5 years. Conversely, GERD-HRQL scores were significantly better in the small-antrectomy group until 24 months; thereafter, results were comparable between groups over the long term. **Conclusions:** Therefore, while wide antrectomy may offer superior short-term anthropometric outcomes, both techniques yield similar long-term results regarding weight management and GERD incidence. Larger prospective studies are needed to further address this issue.

## 1. Introduction

Laparoscopic sleeve gastrectomy (LSG) stands out as the most prevalent primary bariatric procedure globally, renowned for its efficacy, reproducibility and safety in improving anthropometric outcomes [1,2]. Its profound metabolic impact, mediated by significant hormonal pathways, triggers alterations in eating patterns, glycemic regulation, and intestinal dynamics. Despite the widely recognized advantages, the procedure carries inherent risks, including potentially severe complications such as leakage, hemorrhage, splenic injury, stenosis, and gastroesophageal reflux disease (GERD), warranting cautious consideration [3,4,5,6].

While LSG remains a cornerstone in obesity management, it appears difficult to achieve a completely standardized technique. Controversial points include the choice of bougie size, the use of intraoperative leak testing, the configuration of the gastroesophageal junction section, the reinforcement of the staple line, and the distance of resection from the pylorus (i.e., antrectomy), ranging between 2 and 6–7 cm [7,8,9].

Advocates for a greater distance from the pylorus argue its potential benefits in enhancing gastric emptying, averting distal stenosis, and mitigating intraluminal pressure, thereby possibly reducing fistula and reflux occurrences [10,11]. The antrum, in fact, is critical for regulating gastric emptying. The extensive resection of the antrum may alter gastric motility, potentially leading to complications such as GERD. Conversely, proponents of closer resections posit advantages such as reduced gastric capacity and distensibility, heightened intracavitary pressure, and consequent enhanced satiety with reduced oral intake [12,13]. Wide antrectomy may mitigate this risk by maintaining a higher level of restriction over time. This could lead to more durable weight loss outcomes and lower rates of weight regain [13]. Currently, in the literature, only one meta-analysis on this topic is reported by Diab et al. [9]. The authors concluded, based on 627 patients, that antral preservation might shorten length of stay and appears to be associated with lower incidences of prolonged vomiting and GERD in the first few months postoperatively. However, it is associated with decreased weight loss. However, the longest follow-up period of the considered studies was 24 months.

We previously published a preliminary study about this debated topic, concluding that performing LSG with radical antrectomy improved weight loss at the 12-month follow-up, probably decreasing food tolerance compared patients undergoing LSG with limited antrum resection [14]. However, these disparities appeared to diminish by the 24-month follow-up. Additionally, it seemed to contribute to the development of GERD and “de novo” esophagitis. Despite these consistent arguments, comparative long-term investigations into the two approaches remain scarce.

Hence, this retrospective multicentric study aims to elucidate the impact of varying resection distances from the pylorus on weight variation, body mass index (BMI), and percentage of excess weight loss (%EWL) at a 5-year follow-up post-LSG. The study compared subjects undergoing LSG with gastric resections starting 2 cm vs. 6 cm from the pylorus (wide antrectomy versus small antrectomy) and also assesses differences in food tolerance, reflux symptomatology, GERD Health-Related Quality-of-Life (GERD-HRQL) scores, and obesity-related diseases between the two cohorts.

## 2. Methods

This study is reported according to the STROBE statement for cohort studies [15]. A retrospective multicentric study was conducted to assess the surgical and anthropometric outcomes among patients who underwent wide and small antrectomy during LSG. It was conducted according to the ethical principles stated in the Declaration of Helsinki. Written informed consent was obtained from all patients.

### 2.1. Study Setting and Study Population

From 1 January 2015 to 31 December 2019, all patients undergoing LSG for morbid obesity at the Division of General Surgery of a teaching hospital and a tertiary hospital were included in the study. Inclusion criteria were age ≥ 16 years and either a BMI over 40 kg/m^2^ or a BMI ≥ 35 with obesity-related diseases such as type 2 diabetes mellitus (T2DM), hypertension, hyperlipidemia, chronic obstructive pulmonary disease (COPD), osteoarthritis, and degenerative joint disease, according to the Società Italiana di Chirurgia dell’Obesità (SICOB) guidelines [16]. Exclusion criteria were patients undergoing revisional bariatric surgery, the concomitant presence of neoplasms, psychiatric diseases, alcohol dependence, drug addiction, pregnancy or lactation, endocrine disorders causing obesity (such as hypothyroidism and Cushing’s disease), inflammatory bowel disease, Barrett’s esophagus, GERD with esophagitis graded as B or higher, and large hiatal hernias (>3 cm). Additionally, severe GERD, as determined by a GERD Health-Related Quality-of-Life (HRQL) score > 30 and interference with daily activities or dietary restrictions, led to exclusion from the study.

All patients underwent a routine preoperative clinical and instrumental diagnostic assessment, including anamnestic data collection, blood exams, ECG, cardiologic, anesthesiologic, and psychiatric evaluations, thoracic X-ray, pneumologist evaluation with spirometry, complete abdomen and thyroid ultrasound, lower limb Doppler ultrasound, and nutritionist counseling. Evaluation for GERD presence or absence was conducted preoperatively, with the assessment of heartburn and/or regurgitation performed using the GERD HRQL questionnaire. Symptom severity was graded based on the questionnaire scores. Preoperative upper endoscopy (UE) was performed after the discontinuation of proton pump inhibitors (PPIs) or H2 blockers for 20 days, with Helicobacter pylori (HP) testing. HP eradication was performed according to the Maastricht consensus when present. Esophagitis presence was graded using the Los Angeles Classification at UE, and PPI usage and symptom relief were assessed. pH monitoring was indicated in cases of atypical symptoms or PPI refractoriness to confirm or exclude GERD diagnosis.

After the referral for surgery, each patient received a detailed explanation of the procedure from the medical staff and had to sign a personalized informed consent form. All operations were performed by four experienced surgeons who had performed over 250 bariatric procedures. Clinical data were collected in an electronic database and retrospectively analyzed. According to the surgical description reported in the clinical record, patients who underwent wide antrectomy with resection starting at 2 cm from the pylorus were included in Group A, while patients undergoing small antrectomy at 6 cm from the pylorus were assigned to Group B.

### 2.2. GERD Health-Related Quality of Life (HRQL)

Patients received a detailed explanation about each symptom definition and were surveyed about the presence of heartburn and/or regurgitation with a specific questionnaire: the GERD Health-Related Quality of Life (HRQL) [17]. Each of the 10 items of the questionnaire was rated from 0 (absence of symptoms) to 5 (severe symptoms) for a total score ranging from 0 to 50. Symptoms were considered as absent in patients reporting a GERD-HRQL score of 0, mild from 1 to 15, moderate from 16 to 30, and severe from 31 to 50.

### 2.3. Surgical Technique

Pneumoperitoneum was established using our standard technique of Visiport (Medtronic, Dublin, Ireland) positioning at the Palmer site. We obtained access with a standard four-port approach. CO_2_ was insufflated up to 14 mmHg. In patients with a higher body mass index and severe visceral obesity, additional trocars were added for the retraction of the omentum or large fatty livers, optimizing exposure to reach the left crus. Once the left crus was reached, optimal exposure of the hiatus was mandatory to identify incidental hiatal hernias and ensure a complete dissection of the left crus to prevent a retained fundus. The greater omentum was opened close to the stomach wall at a point between the fundus and the antrum to detach the greater curvature completely from the stomach. This dissection started 2 cm proximal to the pylorus and continued along the greater curvature to the left crus in Group A using the blunt tip (Medtronic Inc., Dublin, Ireland), and at 6 cm proximal to the pylorus in Group B. Posterior adhesions, if present, were carefully divided. The left gastrophrenic ligament was divided to expose the angle of His and identify the complete hiatus and fundus. A 36Fr bougie was positioned before starting the stomach resection. The starting point for gastrectomy was 2 cm from the pylorus in Group A (wide antrectomy) and 6 cm from the pylorus in Group B (small antrectomy).

To perform the gastrectomy, we used a 60 mm Echelon Flex^®^ (Ethicon Inc., Raritan, Franklin Township, NJ, USA) stapler. We chose gold cartridges at the antrum level and finished with a blue one. We always checked the posterior wall before firing. Once we reached the proximal stomach, the stapler was positioned 1–2 cm lateral to the left of the angle of His to avoid including esophageal tissue. The staple line was routinely reinforced with an oversewing running suture. Intraoperative endoscopy was used to double-check for intraluminal bleeding and to assess the size and integrity of the staple line. Patients were advised to take lansoprazole 30 mg daily for 6 months and a multivitamin/mineral tablet (Bariatrifast^®^, Bio Italia S.r.l., Rome, Italy) for at least 6 months.

### 2.4. Outcome Measures

Postoperative assessment included the evaluation of anthropometric parameters (including BMI, weight, %Excess Weight Loss [%EWL], and %Total Weight Loss [%TWL]) at 6, 12, 24, 36, and 60 months post-surgery for the entire cohort. Bariatric surgery failure was defined by the onset of insufficient weight loss (IWL) or weight regain (WR). IWL was defined as a loss of less than 50% of the expected excess weight loss (EWL) after bariatric surgery. WR was defined as a weight increase observed after the patient reached their minimum weight (nadir) following bariatric surgery, specifically an increase of 25% of the lost weight compared to the minimum weight achieved, or an increase of more than five BMI points from the nadir [18].

During outpatient follow-up visits, the GERD-HRQL questionnaire was administered to assess gastroesophageal reflux disease symptoms. Postoperative upper endoscopy was conducted throughout the follow-up period based on the onset of GERD symptoms. Notably, significant vomiting and food intolerance were monitored during clinical follow-ups. Significant vomiting was defined as occurring at least three times a week, while food intolerance was characterized by nearly daily vomiting episodes. Esophageal biopsies were performed selectively, specifically in cases of esophagitis graded ≥ B according to the Los Angeles Classification.

During the outpatient visits, a remission/improvement in obesity-related diseases (arterial hypertension, type 2 diabetes, hyperlipidemia, obstructive sleep apnea, and hyperuricemia) was assessed at each visit.

### 2.5. Study Outcomes

The primary outcome of the study focused on comparing anthropometric outcomes, including weight, BMI, %Excess Weight Loss (%EWL), and %Total Weight Loss (%TWL) and bariatric surgery failure (IWL and WR), between the two groups undergoing wide- and small-antrectomy LSG, at a 5-year follow-up. Secondary outcomes included the assessment of incidence of reflux symptoms, GERD Health-Related Quality-of-Life (GERD-HRQL) scores, endoscopical evaluation and obesity-related diseases between the groups.

### 2.6. Statistical Analysis

The population was divided into two groups: patients undergoing wide (Group A) and small (Group B) antrectomy during LSG. Data were described according to each variable type. Continuous variables were expressed as the mean with its standard deviation (SD) or median and range. Frequencies were used for categorical variables. *p*-values below 0.05 were considered significant. Stata 16 (StataCorp, College Station, TX, USA) was utilized for all statistical analyses.

## 3. Results

### 3.1. Study Population

**Figure 1:**
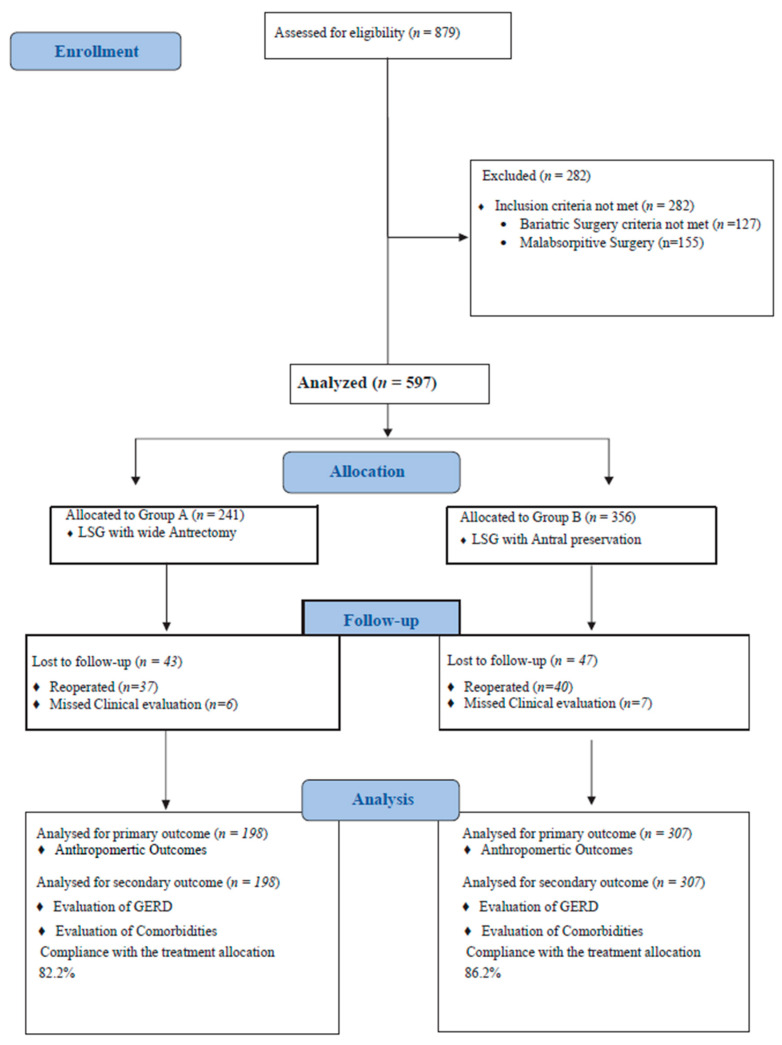
Study flowchart.

Between January 2015 and November 2019, a total of 879 patients referred for severe obesity at our institutions, of whom 752 met the SICOB criteria, received bariatric surgery treatment. Specifically, 241 patients underwent LSG with a starting point 2 cm from the pylorus (Group A), and 356 patients underwent LSG with a starting point 6 cm from the pylorus (Group B) [Figure 1]. In the study population comprising the 597 patients, 315 were females (52.7%) and 282 were males (47.3%), with a mean age of 37.3 ± 7.3 years and a mean weight of 134.7 ± 35.2 kg, a BMI of 44.8 ± 5.2 kg/m2, and Excess Body Weight (EBW) of 71.4 ± 21.9 kg. Baseline demographic and pathological findings are detailed in Table 1. The mean follow-up duration was 69.4 ± 2.2 months.

The mean operative time was 55.7 ± 31.8 min, with no statistical difference between the two groups (Group A: 52.4 ± 25.9 min, Group B: 51.4 ± 29.7 min, *p* = 0.475). The median hospital stay was 5 days for both groups [Group A 5 (4–7) and Group B (4–7), *p* = 0.378]. Intraoperative complications occurred in 4/150 patients (2.6%), including 3 hemorrhage (2 in Group A and 1 in Group B) and bowel injury (Group A), which were managed laparoscopically. There were no 30-day mortalities in either group. Two patients underwent reoperation within 30 days (1.3%)—one in each group—due to bleeding, and both were performed laparoscopically.

At various follow-up intervals, several patients were lost to follow-up, with a drop-out rate at 69.4 ± 2.2 months of 17.8% in Group A (43 patients) and 13.2% (47 patients) in Group B. Overall, 505 patients (84.9%) attended the 5-year follow-up. Twelve patients (9 in Group A and 3 in Group B) underwent RYGB during the follow-up due to an inadequate response to GERD pharmacotherapy and were excluded from the analysis. Twenty-four patients in Group A (9.9%) and 37 patients in Group B (10.4%) patients received a further bariatric procedure for weight regain or insufficient weight loss; these patients were also excluded from the study.

### 3.2. Primary Outcome

Six months post-surgery, significant differences were observed in weight, BMI, %Excess Weight Loss (%EWL), and %Total Weight Loss (%TWL) between the two groups. In Group A, the BMI was 32.3 ± 7.4 compared to 35.9 ± 5.2 in Group B, %EWL was 42.3 ± 7.1 vs. 33.9 ± 7.5, and %TWL was 34.9 ± 9.7 vs. 27.5 ± 9.9 (*p* < 0.05 for each parameter). By 6 months, BMI, %EWL, and %TWL continued to show significant improvements in both groups. In Group A, BMI was 28.13 ± 5.62 compared to 30.1 ± 4.1 in Group B, %EWL was 47.1 ± 12.3 vs. 41.2 ± 10.7, and %TWL was 41.2 ± 7.6 vs. 34.5 ± 8.11 (*p* < 0.05 for each parameter). Similar trends were observed at 12 months, with BMI, %EWL, and %TWL being 24.2 ± 3.4 vs. 27.5 ± 4.3, 63.7 ± 14.1 vs. 59.6 ± 12.5, and 42.9 ± 7.4 vs. 38.2 ± 6.2 in Group A compared to Group B, respectively (*p* < 0.05 for each parameter) [Table 2]. However, from 24 months, no statistically significant differences were recognized in weight, BMI, %EWL, or %TWL between the two groups. Regarding the SG failure, at 60 months, 25 (12.6%) and 38 (12.4%) patients experienced WR in Group A and B, respectively (*p* = 0.934), while IWL was reported in 22 patients (11.1%) and 40 patients (13.1%) in Group A and B, respectively (0.521) [Table 2].

### 3.3. Secondary Outcome

In the preoperative phase, GERD symptoms were present in a minority of patients, with 57 out of 595 (9.6%) presenting GERD symptoms (GERD-HRQL > 16 < 30), evenly distributed between the two groups. In the postoperative period, the incidence of GERD symptoms varied between groups. At 12 months, 61.1% of Group A and 83.9% of Group B reported GERD-HRQL scores < 15 (*p* < 0.001). This trend of fewer severe GERD symptoms in Group B persisted across all time points, with Group B consistently showing significantly better HRQL scores until 24 months (*p* = 0.008). Significant vomiting and food intolerance were more prevalent in Group A at 12 and 24 months but were rare in both groups by the 36- and 60-month follow-ups [Table 3].

Endoscopic assessment was performed in 198 patients (40.7%) from Group A and 187 patients (52.5%) from Group B. The endoscopic findings preoperatively revealed grade A esophagitis in 32 of 595 (5.4%) patients, and hiatal hernias < 2 cm were identified in 31 of 595 (5.2%) patients. No patients reported vomiting or food intolerance preoperatively. Esophagitis was observed postoperatively, with a higher incidence in Group A, although the difference between the groups was not statistically significant. Moreover, esophagitis grade B was significantly higher in Group A until the 36-month follow-up. No cases of Barrett’s esophagus were observed in either group [Table 4].

Regarding the obesity-related diseases, both groups experienced, at all the follow-up points, improvements in all the diseases analyzed (i.e., hypertension, T2DM, hyperlipidemia, obstructive sleep apnea, hyperuricemia), without showing statistical difference between the groups [Table 5].

## 4. Discussion

LSG was actually the first bariatric procedure performed worldwide. It is well accepted by patients, who are often afraid by the irreversibility of the malabsorptive techniques, and also preferred by the surgeons for its safeness and efficacy [19]. Both LSG and laparoscopic RYGB demonstrate remarkable efficacy in terms of %EWL enhancement and comorbidity resolution, with no significant difference between the procedures [20]. LSG’s impact on weight loss is attributed to both restrictive and endocrine mechanisms. Recent research has highlighted reductions in plasma ghrelin levels and alterations in bile acids and FXR signaling post-LSG, contributing to weight loss and improved glucose tolerance [21]. Several large-scale studies have underscored LSG’s advantages, including its technical reproducibility, short operative duration, and immediate caloric restriction. However, the role of gastric pouch volume in weight loss outcomes remains controversial, mirroring the debate surrounding the impact of gastric remnant size in LSG on anthropometric results. Notably, long-term follow-up studies have reported weight loss failure rates up to 25% after LSG, raising questions about its efficacy as a standalone bariatric procedure [22,23]. The causes of such failure remain debated, with some attributing it to the size of the residual stomach. However, conflicting findings were reported, with studies failing to establish a clear correlation between weight loss and the size of the calibration tube used for LSG [24,25]. Currently, experts are adopting larger bougies to minimize strictures and leak risks, typically starting the resection approximately 3–4 cm from the pylorus. Moreover, despite LSG being the most-performed bariatric procedure worldwide, in fact, several reports have analyzed its long-term results, focusing on the onset of postoperative GERD, severe reflux and Barrett’s esophagus, and produced worrying results [26,27]. Therefore, along with the use of fundoplication associated to conventional LSG, the so-called F-Sleeve, the surgical community prompted studies to define technical aspects able to reduce these fearsome complications, such as antrum preservation [4,28].

Some studies, in fact, have suggested better weight loss outcomes with radical antrectomy, while others have linked smaller gastric tubes to higher digestive intolerance and severe reflux [9,29,30,31]. In detail, Obeidat et al. [32] assigned 54 patients to a group receiving LSG 6 cm proximal to the pylorus and 56 patients to a group receiving LSG 2 cm proximal to the pylorus with partial antrectomy. The antrectomy group showed significantly higher weight loss at 12 and 24 months, concluding that LSG with antrectomy not only resulted in greater weight loss but also safely enhanced the restrictive effect of LSG. Similarly, ElGeidie et al. found significantly higher weight loss in the antrectomy group at 6 months, but this difference was not significant at 12 months [31]. Conversely, our study found significantly higher weight loss in the antrectomy group only at 3 months, with no significant difference at 6, 12, and 24 months. A recent metanalysis at a 24-month follow-up concluded that when compared to a 2 cm distance from the pylorus (antral resection), a 6 cm distance from the pylorus (antral preservation) may reduce the length of hospital stay and be associated with a lower incidence of prolonged vomiting and GERD in the initial months postoperatively. However, it is also linked to decreased weight loss [9]. We previously reported our pilot analysis on this topic in a monocentric prospective study, comparing the outcomes of wide antrectomy versus small antrectomy during LSG in 150 patients, concluding that radical antrectomy allowed faster weight loss in the first 12 months, but the differences were no longer identifiable at 24 months [14]. Moreover, the authors concluded that wide antrectomy was associated with more severe GERD and food intolerance [14].

Considering the remarkable results of the previous study and the lack of studies with long-term follow-up, we analyzed the anthropometric outcomes and the surgical outcomes, including GERD onset and obesity-related disease resolution, in patients undergoing small (6 cm from pylorus) versus wide (2 cm from pylorus) antrectomy at a 60-month follow-up. To the best of our knowledge, the current study is the largest analyzing this topic with such a long follow-up.

In the current series, we found significant differences in weight, BMI, %EWL and %TWL, favoring Group A (wide antrectomy) until 12 months post-surgery (*p* < 0.05). Subsequently, the two groups did not show any statistical differences. Noteworthily, all the anthropometric parameters slightly inverted the improving trend between 36 and 60 months, probably due to cases of WR (12.6% in Group A vs. 12.4% in Group B, *p* = 0.934).

Patients in Group A exhibited a higher incidence of vomiting and food intolerance in the first 24 months, which gradually decreased by the 36-month follow-up. At 12 and 24 months, Group B had significantly better GERD HRQL scores; by 36 and 60 months, the differences between the groups in GERD HRQL scores become less pronounced and were not statistically significant, suggesting a convergence in GERD outcomes over time. Conversely, severe symptoms (GERD HRQL > 31) remained significantly higher in patients with wide antrectomy until 36 months after surgery.

Regarding the endoscopic findings, no significant differences were observed in esophagitis A at any timepoint between the groups. However, Group A had higher rates of esophagitis B at 12, 24, and 36 months, with statistically significant differences.

Wide and small antrectomy resulted similar in the long-term management of the obesity-related diseases. Both groups, in fact, showed significant reductions in the prevalence of T2DM, hypertension, hyperlipidemia, obstructive sleep apnea, or hyperuricemia at any timepoint, results similar to the literature benchmark. These results appeared different from some reports that highlighted a higher rate of T2DM in patients undergoing wide antrectomy [33,34] Noteworthily, both reports analyzed patients at 12- and 24-month follow-ups [33,34].

Therefore, wide antrectomy (Group A) demonstrated greater initial weight loss and BMI reduction, probably due to the higher rate of food intolerance, but these advantages diminished over time for a physiologic adaptation and, probably, for the initial enlargement of the gastric pouch. Conversely, the GERD symptoms seemed to afflict this group for longer. Group B (small antrectomy) had better outcomes concerning GERD symptoms and HRQL scores, suggesting a trade-off between weight loss efficacy and gastroesophageal symptom management. The preoperative selection of patients is crucial, considering the potential risks associated with small antrum size, including GERD and esophagitis.

The current study has several limitations to address. First is the retrospective design of the study. Moreover, the majority of patients were tested for GERD symptoms only with the GERD HRQL score and not objectively evaluated, since the endoscopy was performed in 198 patients (40.7%) in Group A and 187 patients (52.5%) in Group B.

## 5. Conclusions

Performing LSG with radical antrectomy may enhance and expedite weight loss at the 12-month follow-up but compromise food tolerance compared to LSG with limited antrum resection. However, these differences appear to diminish by the 24-month follow-up. Additionally, a smaller antrum may predispose patients to GERD and de novo esophagitis. Therefore, meticulous preoperative patient selection is recommended. Larger prospective studies are needed to address this issue and to highlight the incidence of each variable in the future weight loss of bariatric patients undergoing LSG.

## Figures and Tables

**Table 1:** Preoperative demographics data. BMI body mass index, T2MD type 2 mellitus diabetes, COPD chronic obstructive pulmonary disease. * Values are mean ± standard deviation.

	Group A (241 pts)	Group B (356 pts)	*p*
Age (years) *	37.1 ± 6.7	36.8 ± 7.9	0.149
Gender (female/male)	131/110	184/172	0.521
Weight (kg) *	135.7 ± 11.4	134.4 ± 9.5	0.489
BMI (kg/m^2^) *	45.1 ± 3.8	44.3 ± 9.8	0.612
ASA (I–II) (%)	180 (74.6%)	273 (76.7%)	0.575
ASA (III–IV) (%)	61 (25.4%)	83 (23.3%)	0.575
T2MD (%)	31 (12.8%)	42 (11.8%)	0.696
COPD (%)	6 (2.5%)	9 (2.5%)	0.976
Heart ischemia (%)	0	1 (0.2%)	0.411
Hypertension (%)	159 (65.9%)	225 (63.2%)	0.487

**Table 2:** Anthropometric outcomes of the two groups at the different follow-up periods. BMI body mass index, %EWL percentage excess weight loss, %TWL percentage total weight loss. * Values are mean ± standard deviation.

	6 Months			12 Months			24 Months			36 Months			60 Months		
	Group A (241 pts)	Group B (354 pts)	*p*	Group A (226 pts)	Group B (331 pts)	*p*	Group A (218 pts)	Group B (321 pts)	*p*	Group A (210 pts)	Group B (314 pts)	*p*	Group A (198 pts)	Group B (307 pts)	*p*
Weight (kg) *	93.3 ± 6.4	98.4 ± 7.2	<0.05	87.4 ± 6.9	90.7 ± 4.9	<0.05	78.8 ± 7.8	79.5 ± 6.4	0.128	79.8	80.3	0.423	88.5 ± 5.9	90.1 ± 6.1	0.345
BMI (kg/m^2^) *	32.3 ± 7.4	35.9 ± 5.2	<0.05	28.1 ± 3.7	30.5 ± 4.1	<0.05	26.7 ± 6.3	27.3 ± 4.7	0.348	27.1 ± 3.8	27.9 ± 6.9	0.486	29.1 ± 4.9	29.9 ± 5.2	0.215
%EWL	66.3 ± 7.1	62.9 ± 7.5	<0.05	70.7 ± 5.6	67.3 ± 7.8	<0.05	79.7 ± 8.2	78.9 ± 7.4	0.245	78.4 ± 4.7	77.6 ± 5.9	0.348	70.1 ± 8.2	68.9 ± 5.8	0.157
%TWL	31.9 ± 9.7	27.5 ± 9.9	<0.05	32.9 ± 7.5	30.1 ± 4.9	<0.05	36.8 ± 7.9	35.7 ± 7.3	0.318	35.7 ± 9.7	34.8 ± 7.2	0.319	34.7 ± 6.9	33.2 ± 5.8	0.229
Weight regain	0	0	-	0	0	-	12 (5.5%)	19 (5.9%)	0.839	21 (10%)	33 (10.5%)	0.850	25 (12.6%)	38 (12.4%)	0.934
Insufficient weight loss	1 (0.4%)	2 (0.6%)	0.799	4 (1.8%)	7 (2.1%)	0.773	9 (4.1%)	22 (6.8%)	0.146	18 (8.6%)	36 (11.5%)	0.285	22 (11.1%)	40 (13.1%)	0.521

**Table 3:** GERD Health-Related Quality-of-Life (GERD-HRQL) preoperative and postoperative scores. GERD-HRQL (GERD Health-Related Quality-of-Life).

	Preoperative			12 Months			24 Months			36 Months			60 Months		
	Group A (241 pts)	Group B (354 pts)	*p*	Group A (226 pts)	Group B (331 pts)	*p*	Group A (218 pts)	Group B (321 pts)	*p*	Group A (210 pts)	Group B (314 pts)	*p*	Group A (198 pts)	Group B (307 pts)	*p*
GERD HRQL < 15	217 (90.4%)	321 (90.6%)	0.914	138 (61.1%)	278 (83.9%)	<0.001	175 (80.3%)	291(90.7%)	0.008	191 (90.9%)	301 (95.8%)	0.211	188 (94.9%)	299 (97.4%)	0.148
GERD HRQL > 16 < 30	24 (9.9%)	33 (90.4%)	0.795	68 (30.1%)	51 (15.5%)	<0.001	28 (12.8%)	28 (8.7%)	0.123	15 (7.2%)	11 (3.6%)	0.060	7 (3.6%)	8 (2.6%)	0.548
GERD HRQL > 31	-	-	-	20 (8.8%)	2 (0.6%)	<0.001	15 (6.9%)	2 (0.6%)	<0.001	6 (2.9%)	2 (0.6%)	0.042	3 (1.5%)	0	0.334
Significant vomiting	-	-	-	8 (3.5%)	1 (0.3%)	0.002	5 (2.3%)	0	0.006	1 (0.5%)	0	0.221	1 (0.5%)	0	0.212
Intolerance to food	-	-	-	2 (0.9%)	0	0.035	0	0	-	0	0	-	0	0	-

**Table 4:** Preoperative and follow-up upper endoscopy findings in the two groups.

	Preoperative			12 Months			24 Months			36 Months			60 Months		
	Group A (241 pts)	Group B (356 pts)	*p*	Group A (198 pts)	Group B (287 pts)	*p*	Group A (162 pts)	Group B (258 pts)	*p*	Group A (122 pts)	Group B (229 pts)	*p*	Group A (98 pts)	Group B (187 pts)	*p*
Esophagitis A	11 (4.5%)	21 (5.9%)	0.477	22 (11.1%)	24 (8.4%)	0.503	17 (10.5%)	21 (8.1%)	0.413	16 (13.1%)	22 (9.8%)	0.313	11 (11.2%)	16 (8.6%)	0.465
B	-	-	-	12 (6.1%)	3 (1.1%)	0.001	9 (5.6%)	2 (0.8%)	0.002	6 (4.1%)	1(0.4%)	<0.001	3 (3.1%)	1 (0.5%)	0.085
C	-	-	-	2 (1%)	-	0.088	1 (0.6%)	-	0.206	1 (0.8%)	-	0.170	1 (1%)	-	0.166
D	-	-	-	-	-	-	-	-	-	-	-	-	-	-	
Metaplasia	-	-	-	-	-	-	-	-	-	-	-	-	-	-	-
Helicobacter Pylori	22 (9.1%)	29 (8.1%)	0.673	-	-	-	-	-	-	-	-	-	-	-	-
Hiatal Hernia < 2 cm	13 (5.4%)	18 (5.1%)	0.855	12 (6.1%)	16 (5.6%)	0.821	9 (5.6%)	12 (4.6%)	0.678	9 (7.3%)	11 (4.8%)	0.321	6 (6.1%)	9 (4.8%)	0.638

**Table 5:** Preoperative and follow-up obesity-related diseases in the two groups.

	Preoperative			12 Months			24 Months			36 Months			60 Months		
	Group A (241 pts)	Group B (354 pts)	*p*	Group A (226 pts)	Group B (331 pts)	*p*	Group A (218 pts)	Group B (321 pts)	*p*	Group A (210 pts)	Group B (314 pts)	*p*	Group A (198 pts)	Group B (307 pts)	*p*
T2DM	51 (21.2%)	72 (20.3%)	0.807	22 (9.7%)	34 (10.2%)	0.835	15 (6.9%)	24 (7.4%)	0.793	12 (5.7%)	21 (6.7%)	0.653	15 (7.6%)	22 (7.2%)	0.863
Hypertension	159 (65.9%)	225 (63.6%)	0.545	45 (19.9%)	70 (21.1%)	0.723	22 (10.1%)	34 (10.6%)	0.851	14 (6.6%)	22 (7.1%)	0.880	17 (8.6%)	25 (8.1%)	0.860
Hyperlipidemia	157 (65.1%)	223 (62.9%)	0.591	42 (18.5%)	65 (19.6%)	0.756	21 (9.6%)	32 (9.9%)	0.897	16 (7.6%)	28 (8.9%)	0.599	24 (12.1%)	32 (10.4%)	0.553
Obstructive Sleep Apnea	113 (46.8%)	169 (47.8%)	0.838	41 (18.1%)	57 (17.2%)	0.742	31 (14.2%)	46 (14.3%)	0.971	14 (6.6%)	25 (7.9%)	0.199	12 (6.1%)	22 (7.2%)	0.628
Hyperuricemia	37 (15.3%)	49 (13.8%)	0.606	22 (9.7%)	31 (9.4%)	0.884	7 (3.2%)	12 (3.7%)	0.744	1 (0.5%)	1 (0.3%)	0.774	0 (0%)	1 (0.3%)	0.421

## Data Availability

The datasets used and/or analyzed during the current study are available from the corresponding author on reasonable request.
